# Icariin interacts with IGFBP3 to alleviate diabetic cataract through PI3K/AKT signaling pathway

**DOI:** 10.1016/j.isci.2025.112796

**Published:** 2025-05-30

**Authors:** Yakun Wang, Wenxian Yang, Hangjia Zuo, Shuhao Zeng, Xianyang Liu, Fan Cao, Hui Yang, Shangze Gao, Meng Tian, Xiang Gao, Yongguo Xiang, Fanfan Huang, Baorui Chu, Chao Wu, Hui Feng, Wenjuan Wan, Shijie Zheng, Shengping Hou, Ke Hu

**Affiliations:** 1 The First Affiliated Hospital of Chongqing Medical University, Chongqing Key Laboratory of Prevention and Treatment on Major Blinding Diseases, Chongqing Eye Institute, Chongqing Branch (Municipality Division) of National Clinical Research Center for Ocular Diseases, Chongqing 400016, China; 2 Beijing Institute of Ophthalmology, Beijing Tongren Eye Center, Beijing Tongren Hospital, Capital Medical University, Beijing Ophthalmology & Visual Sciences Key Laboratory, Beijing 100730, China

**Keywords:** Medicine, Molecular biology, Molecular medicine

## Abstract

Diabetic cataract (DC), a well-recognized complication in diabetic patients, can progress to blindness if not adequately managed, with currently limited therapeutic strategies. Icariin (ICA), a natural compound derived from Epimedium, has been demonstrated exhibiting anti-inflammatory and anti-oxidant, but its impact on diabetic cataracts remains elusive. In this study, we used both *in vitro* SRA01/04 cells and *in vivo* SD rats’ model to explored the protective effects of ICA in cataract formation. Following network pharmacology, proteomic and surface plasmon resonance (SPR) analyses further demonstrated that ICA interacts with insulin-like growth factor-binding protein-3 (IGFBP3) and modulates oxidative stress as well as apoptosis via the PI3K/AKT signaling pathway. These findings collectively demonstrated that ICA could alleviate high glucose-induced oxidative stress and cell apoptosis *in vitro* and *in vivo*, suggesting that ICA is a potent natural compound with protective effects in DC, offering an effective therapeutic approach for the disease management.

## Highlights

• Icariin alleviates diabetic cataract of streptozotocin-induced SD rats
• Icariin alleviates high glucose-induced oxidative stress and cell apoptosis
• IGFBP3 may be a potential biomarker in diabetic cataract
• Icariin binds to IGFBP3 enhancing PI3K/AKT signaling pathway

## Graphical abstract

**Figure:**
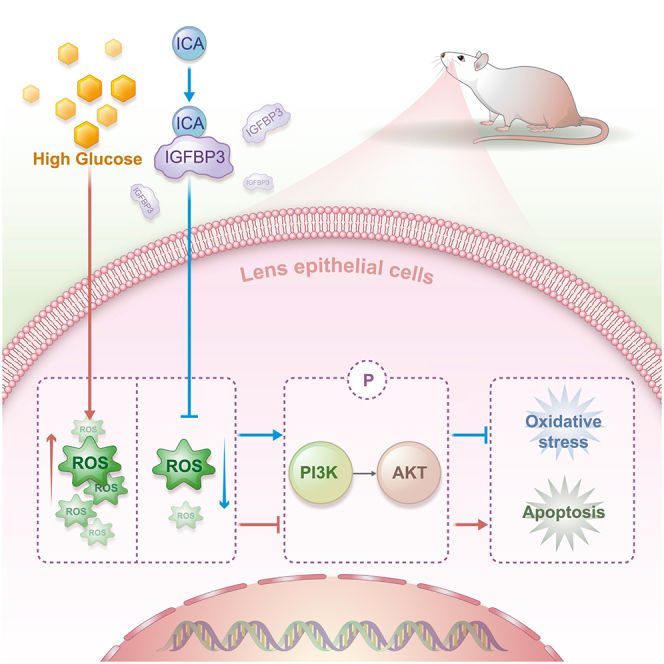

## Introduction

Diabetic cataract is a common ocular complication of diabetes, which can cause vision impairment and blindness in diabetic patients.^1^ Diabetic patients tend to develop cataracts earlier and experience faster progression compared to non-diabetic individuals.^2^ Multiple mechanisms are involved in the formation of diabetic cataracts.^3^^,^^4^ Recent studies have revealed that oxidative stress and cell apoptosis would increase in the lens when exposed to a high blood sugar environment, contributing to the development of cataracts.^5^^,^^6^ Oxidative stress is primarily mediated by reactive oxygen species (ROS), which main include superoxide radical, peroxide, and hydroxyl radical.^7^ Elevated levels of ROS or a reduction in antioxidant capacity both exacerbate oxidative stress, perturb the intracellular milieu, and trigger the apoptotic cascade in lens epithelial cells (LECs), ultimately leading to cataract formation.^8^^,^^9^^,^^10^ Enhancing the antioxidant and anti-apoptotic capabilities of LECs could represent a viable therapeutic approach to mitigate cataract development. Therefore, oxidative stress and cell apoptosis play a crucial role in the pathogenesis of diabetic cataracts.^11^^,^^12^

It is estimated that around 50% of cataract surgeries may be unnecessary if the development of cataracts is delayed by approximately 10 years, in terms of diabetic cataract, if it could be well controlled.^13^ Therefore, finding a compound that can slow down the progression of cataracts is necessary.

Icariin (ICA), a flavonoid compound extracted from the leaves and stems of Epimedium, possesses various pharmacological and biological functions, such as anti-inflammatory, anti-diabetic, antioxidant, anti-sexual dysfunction, anti-atherosclerosis, anti-asthma, and anti-menstrual disorders.^14^^,^^15^^,^^16^ Previous research has shown that ICA can effectively treat and alleviate the occurrence and progression of uveitis and diabetic retinopathy.^17^^,^^18^ However, it is currently unclear whether the protective effects of ICA on diabetic cataracts are mediated through inhibiting oxidative stress or apoptotic mechanisms.

Recent advances in systems biology—including network pharmacology, proteomics, and SPR—have enhanced our understanding of ICA-target interactions and signaling pathways. Network pharmacology maps molecular interactions, proteomics quantifies protein expression, and surface plasmon resonance (SPR) reveals binding kinetics. Collectively, these methods provide a robust framework for elucidating the molecular mechanisms underlying ICA’s protective effects.

In this study, we investigated whether ICA can alleviate the development of diabetic cataracts by mitigating oxidative stress and apoptosis in LECs, and we further explored whether these protective effects are mediated through the PI3K/AKT signaling pathway. The outcomes of our research are anticipated to provide attractive insights and promising strategies for the therapeutic management of diabetic cataracts.

## Results

### Icariin mitigates diabetic cataract progression in SD rats

**Figure 1:**
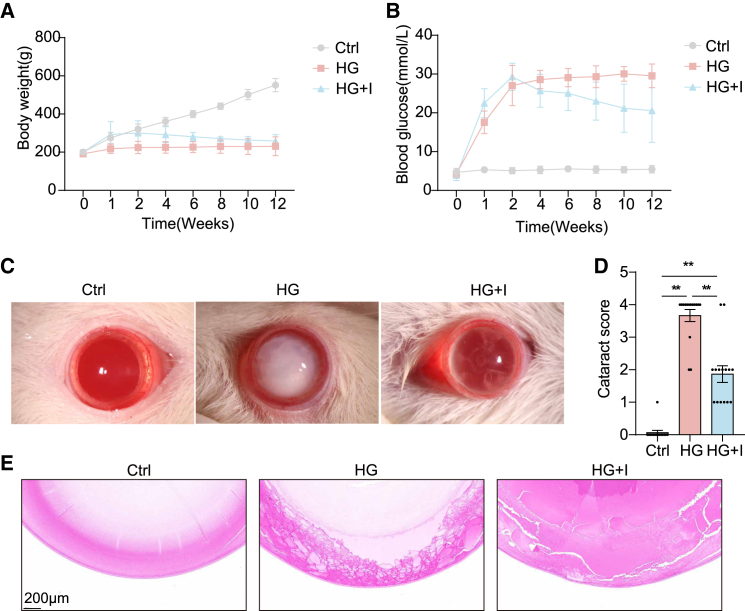
ICA alleviates diabetic cataracts in diabetic SD rats (A) Measurements of changes in body weight in SD rats in no-diabetic rats treated with normal saline (Ctrl), diabetic rats treated with normal saline (HG), and diabetic rats treated with normal saline + ICA (HG + I) during 12 weeks of continuous feeding. (B) Measurements of changes in blood glucose in SD rats in Ctrl, HG, and HG + I groups during 12 weeks of continuous feeding. (C) Photograph of the lens of rats in Ctrl, HG, and HG + I groups. (D) Statistic analysis of (C). (E) Hematoxylin and eosin (H&E) stains of eyes in diabetic rats in Ctrl, HG, and HG + I groups, Scale bars, 200 μm. Data are representative of three independent experiments. The data are presented as the mean ± SEM. *n* = 15. ∗*p* < 0.05 and ∗∗*p* < 0.01.

Over a 12-week period post-diabetes induction, body weight and blood glucose levels were monitored across all experimental groups. In the control group, blood glucose remained within the normal range, whereas rats in both the high glucose (HG) and high glucose plus icariin (HG + I) groups exhibited hyperglycemia (≥16.7 mmol/L). Additionally, control rats showed a continuous increase in body weight, while the HG and HG + I groups maintained relatively stable weights (Figures 1A and 1B).

Following 12 weeks of daily oral administration of ICA (20 mg/kg), its presence was confirmed in the aqueous humor (Figure S1B). Subsequent assessments revealed significant improvements in cataract grading (Figures 1C and 1D) and a reduction in pathological scores (Figure 1E). Collectively, these findings indicate that ICA treatment delays and mitigates the development of diabetic cataracts in this rat model.

### ICA alleviates high glucose-induced cellular oxidative stress and apoptosis

**Figure 2:**
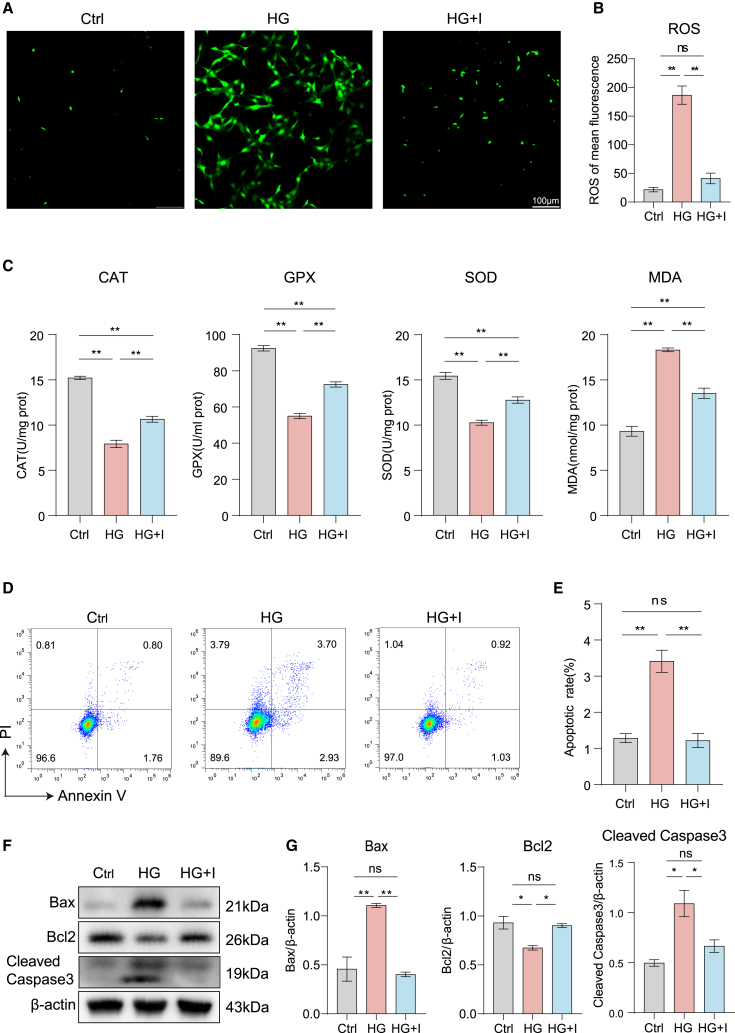
ICA alleviates high glucose-induced oxidative stress and apoptosis in SRA01/04 cells (A) The level of ROS in SRA01/04 cells stimulated by DMEM (Ctrl), high glucose DMEM (HG), and high glucose DMEM + ICA (HG + I) for 48 h, Scale bars, 100 μm. (B) Statistic analysis of (A). (C) The levels of CAT, GPx, SOD, and MDA in SRA01/04 cells by Ctrl, HG, and HG + I treatment for 48 h. (D) Flow cytometry was performed to analyze the effect in SRA01/04 cells by Ctrl, HG, and HG + I treatment for 48 h. (E) Statistic analysis of (D). (F) Immunoblot analysis of the apoptosis-related proteins in SRA01/04 cells by Ctrl, HG, and HG + I treatment for 48 h. (G) Statistic analysis of (F). Data are representative of three independent experiments. The data are presented as the mean ± SEM. *n* = 3. ∗*p* < 0.05 and ∗∗*p* < 0.01.

ICA has been shown to be effective in the treatment of diabetes and its complications.^19^^,^^20^ However, its specific mechanism in diabetic cataract is still unclear. To explore the effect of ICA on oxidative stress in SRA01/04 cells, initially, we demonstrated that 20 μM is the optimal concentration of ICA in stimulating SRA01/04 cells (Figure S2). Then, the accumulation of ROS in SRA01/04 cells was measured using the fluorescent probe DCFH-DA. The fluorescence intensity of ROS in the high glucose group cells significantly increased; however, those treated with ICA showed a significant decrease (Figures 2A and 2B). Meanwhile, we detected the level of catalase (CAT), glutathione peroxidase (GPx), superoxide dismutase (SOD), and malondialdehyde (MDA), which are the indicators reflecting the level of oxidative stress. Data showed that high glucose increased the level of CAT, GPx, and SOD and decrease the level of MDA. ICA could improve the effects of high glucose on CAT, GPx, SOD, and MDA (Figure 2C). These results indicated that ICA could inhibit high glucose-induced oxidative stress in SRA01/04 cells.

Lens opacity in cataracts is primarily attributed to crystalline protein degradation, protein misfolding, or sorbitol accumulation in diabetic conditions. Additionally, cell apoptosis contributes to lens opacity in diabetic cataracts. Then we observed the influence of ICA on high glucose-induced apoptosis in SRA01/04 cells by flow cytometry. Data showed that ICA treatment significantly decreased the rate of late apoptosis induced by high glucose in SRA01/04 cells (Figures 2D and 2E). Furthermore, we found that ICA could reduce Bax and caspase-3 expression and increase Bcl2 expression in high glucose-induced SRA01/04 cells (Figure 2F). These results indicated that ICA exerts a protective effect on oxidative stress and cell apoptosis in high glucose-induced SRA01/04 cells.

### ICA mediates oxidative stress protection through PI3K/AKT pathway

**Figure 3:**
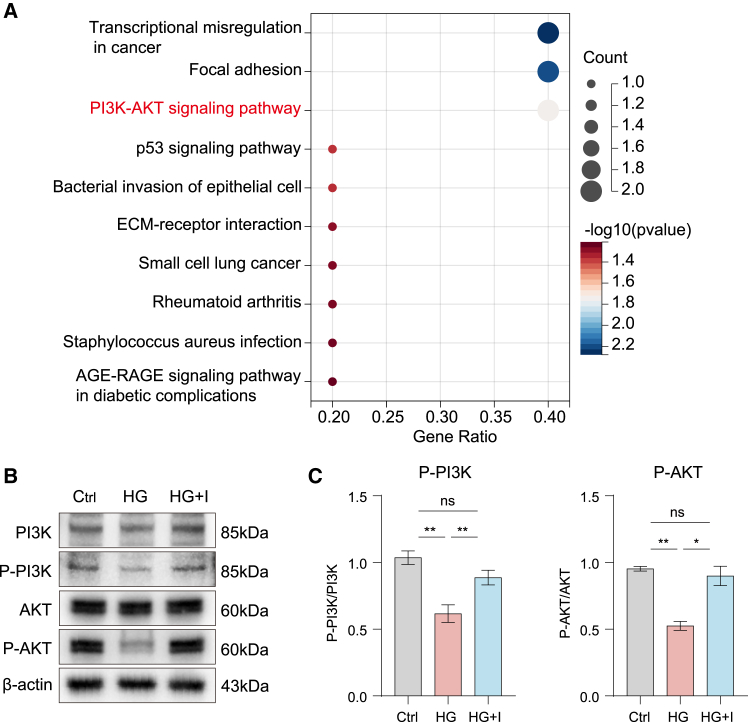
ICA enhances the PI3K/AKT signal pathway in SRA01/04 cells (A) Kyoto Encyclopedia of Genes and Genomes (KEGG) pathway analysis of the differentially expressed proteins upregulated in the HG + I group compared to the HG group. (B) Immunoblot analysis of the indicated proteins in SRA01/04 cells stimulated by DMEM (Ctrl), high glucose DMEM (HG), and high glucose DMEM + ICA (HG + I) for 48 h. (C) Statistic analysis of (B). Data are representative of three independent experiments. The data are presented as the mean ± SEM. *n* = 3. ∗*p* < 0.05 and ∗∗*p* < 0.01.

To investigate the pathways through which ICA mediates its therapeutic effects under hyperglycemic conditions, we employed an integrated approach combining network pharmacology and proteomic profiling. Kyoto Encyclopedia of Genes and Genomes (KEGG) pathway enrichment analysis of the multi-omics data identified the ten most significantly enriched signaling pathways (Figure 3A; Figures S3A–S3C). We focused on the PI3K/AKT signaling pathway, a classic pathway involved in phosphatidylinositol metabolism and mediated by receptor tyrosine kinase. This pathway plays a critical role in regulating oxidative stress and apoptosis. It plays an important role in oxidative stress and apoptosis. Western blotting analysis revealed that ICA treatment enhanced the phosphorylation of PI3K and AKT, which decreased by high glucose in SRA01/04 cells (Figures 3B and 3C). These results suggested that ICA enhances the PI3K/AKT signaling pathway in high glucose-induced SRA01/04 cells.

### Inhibition of the PI3K/AKT pathway reversed the protective effect of icariin

**Figure 4:**
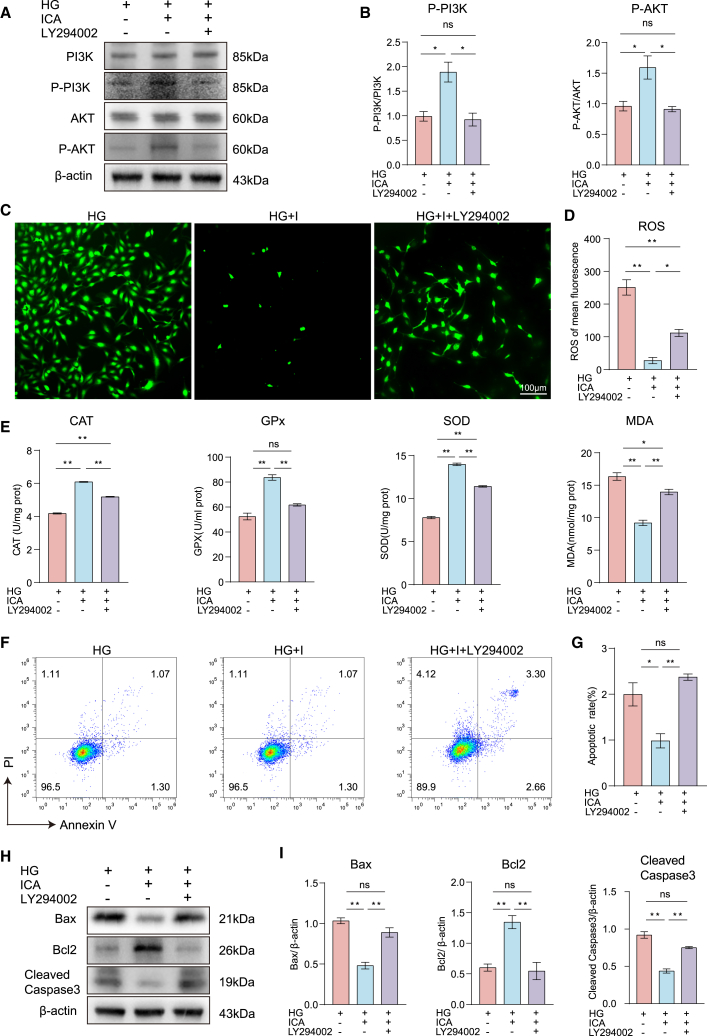
Inhibiting the PI3K/AKT pathway can reverse the protective effect of icariin on SRA01/04 cells (A) Immunoblot analysis of the indicated proteins in SRA01/04 cells stimulated by high glucose DMEM (HG), high glucose DMEM + ICA (HG + I), and high glucose DMEM + ICA + LY294002 (HG + I + LY294002) for 48 h. (B) Statistic analysis of (A). (C) The level of ROS in SRA01/04 cells stimulated by HG, HG + ICA, and HG + I + LY294002 for 48 h, Scale bars, 100 μm. (D) Statistic analysis of (C). (E) The levels of CAT, GPx, SOD, and MDA in SRA01/04 cells stimulated by HG, HG + I, and HG + I + LY294002 for 48 h. (F) Flow cytometry was performed to analyze the effect in SRA01/04 cells stimulated by HG, HG + ICA, and HG + ICA + LY294002 for 48 h. (G) Statistic analysis of (F). (H) Immunoblot analysis of the apoptosis-relative proteins in SRA01/04 cells stimulated by HG, HG + I, and HG + I + LY294002 for 48 h. (I) Statistic analysis of (H). Data are representative of three independent experiments. The data are presented as the mean ± SEM. *n* = 3. ∗*p* < 0.05 and ∗∗*p* < 0.01.

Then we further verified that ICA improved diabetic cataracts through the PI3K/AKT signaling pathway by compound LY294002, which is the inhibitor of PI3K. Western blotting analysis showed that inhibitor LY294002 could block the phosphorylation of PI3K and AKT enhanced by ICA in high glucose-induced SRA01/04 cells (Figures 4A and 4B). Moreover, we found that LY294002 reversed the decrease of ROS induced by ICA (Figures 4C and 4D). Furthermore, we found that the levels of CAT, GPx, and SOD significantly decreased, while the level of MDA significantly increased after inhibitor LY294002 treatment (Figure 4E). These results demonstrate that LY294002 reverses ICA’s antioxidative effects by inhibiting the PI3K/AKT pathway. In addition, we verified the impact of the PI3K/AKT pathway inhibition on cell apoptosis decreased by ICA. Flow cytometry revealed that apoptosis was increased by inhibitor LY294002 treatment (Figures 4F and 4G). Western blotting analysis showed that LY294002 increased the expression of Bax and caspase-3 while decreased the expression of Bcl2 in high glucose-induced SRA01/04 cells (Figures 4H and 4I). In conclusion, the PI3K inhibitor LY294002 can reverse the antioxidant and anti-apoptotic effects of ICA on SRA01/04 cells under high glucose stimulation by inhibiting the PI3K/AKT pathway.

### ICA interacts with IGFBP3 to activate the PI3K/AKT signaling pathway

**Figure 5:**
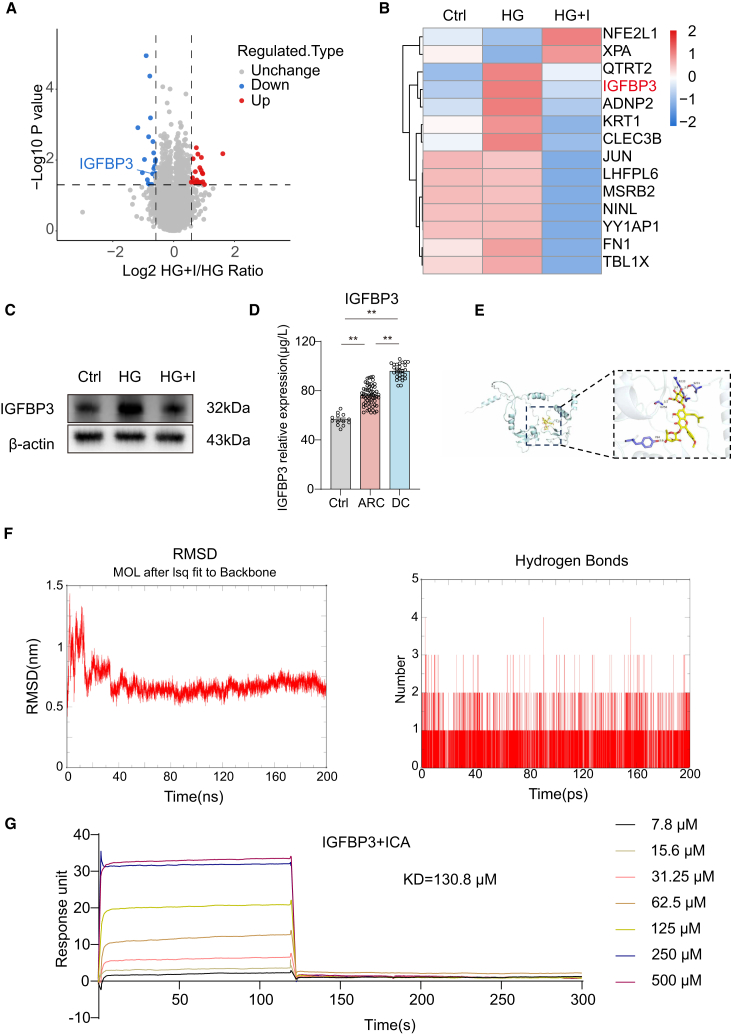
IGFBP3 is a target for ICA (A) Volcano plot (HG + I vs. HG) of differentially expressed proteins; proteins enriched in signaling pathways (upregulated and downregulated) are labeled. (B) Heatmap shows protein expression in every group. (C) Immunoblot analysis of the IGFBP3 in SRA01/04 cells stimulated by DMEM (Ctrl), high glucose DMEM (HG), and high glucose DMEM + ICA (HG + I) for 48 h. (D) IGFBP3 expression in aqueous humor from patients with transparent crystalline lens (Ctrl, *n* = 13, female = 7, male = 6), age-related cataract (ARC, *n* = 59, female = 42, male = 17), and diabetic cataract (DC, *n* = 28, female = 17, male = 11). (E) The hydrogen bond between ICA and IGFBP3 are shown by yellow dashed lines, and the residues (blue) of the interactions and Icariin (yellow) are displayed as sticks. (F) At 40–70 ns, the average RMSD is 0.4906 and the standard deviation is 0.0488; analysis of hydrogen bond formation in the ICA and IGFBP3 complex. (G) The affinity and binding kinetics of ICA for IGFBP3 measured by SPR. Serial dilutions (2-fold) from 7.8 μM compounds in injected into the captured IGFBP3 protein. Kinetic data from one representative experiment were fit to a 1:1 binding model. The profiles are shown for IGFBP3. Data are representative of three independent experiments. The data are presented as the mean ± SEM. *n* = 3. ∗*p* < 0.05 and ∗∗*p* < 0.01.

To further investigate how ICA exerted its effects through the PI3K/AKT pathway, proteomic analysis was conducted on SRA01/04 cells from different treatment groups. Utilizing stringent quality criteria, the proteomic profiling of SRA01/04 cells resulted in the identification of a substantial number of peptides and proteins. Specifically, 63,853 peptides, encompassing 62,903 unique entities, were characterized. Concurrently, 7,996 distinct proteins were discerned, with 7,966 of these proteins being quantifiable. Within this proteomic landscape, a notable subset of proteins exhibited significant alterations in expression levels. In the comparative analysis between the HG + I and HG groups, 20 proteins were observed to be upregulated, while 21 proteins were downregulated, highlighting the differential expression patterns between these two conditions (Figure 5A). After integrating the results of network pharmacology and proteomics, we found that among the downregulated proteins, the expression trend of IGFBP3 correlated with the PI3K/AKT pathway (Figures 5B and S3C). Subsequently, we confirmed IGFBP3 expression via western blotting analysis and further validated its functional interaction with PI3K using computational protein-protein docking, demonstrating mechanistic consistency with proteomics-derived interaction profiles (Figure 5C; Figure S4). Subsequently, we extended our investigation to human subjects. Utilizing an IGFBP3 ELISA test kit, we assessed the levels of IGFBP3 in the aqueous humor of individuals. Our findings indicated that the concentration of IGFBP3 was significantly elevated in aqueous humor of diabetic cataract patients compared to age-related cataract patients and control group, underscoring a potential biomarker for this ocular condition (Figure 5D).

To investigate the binding characteristics of ICA to IGFBP3, the molecular docking studies, which gave deep insight into the binding pattern between ICA and IGFBP3, were performed. The small molecule (yellow) and the amino acid residues involved in hydrogen bond interactions (blue) were displayed in the stick model, with red dashed lines indicating hydrogen bonds. The numbers on the hydrogen bonds indicated the lengths of the hydrogen bonds. The amino acid residues that interacted with hydrogen bonds with the small molecule and protein were Y84, S231, R233, and G256, with bond lengths of 2.2 Å, 2.0 Å, 3.4 Å, and 3.1 Å, respectively (Figure 5E). Further, we carried out dynamic simulations between ICA and IGFBP3, root-mean-square deviation (RMSD) and hydrogen bonds analysis showed that their interaction was stable (Figure 5F). Furthermore, SPR was employed to assess the affinity of ICA to IGFBP3. IGFBP3 was immobilized on the surface of Biacore Chip CM5. Then, various concentrations of compounds were prepared and injected to pass over the surface. The equilibrium dissociation constant (K_D_) value for IGFBP3 against ICA was 130.8 μM (Figure 5G). These data illustrated that ICA could bind to IGFBP3 stably.

### ICA alleviates oxidative stress and apoptosis in diabetic SD rats

**Figure 6:**
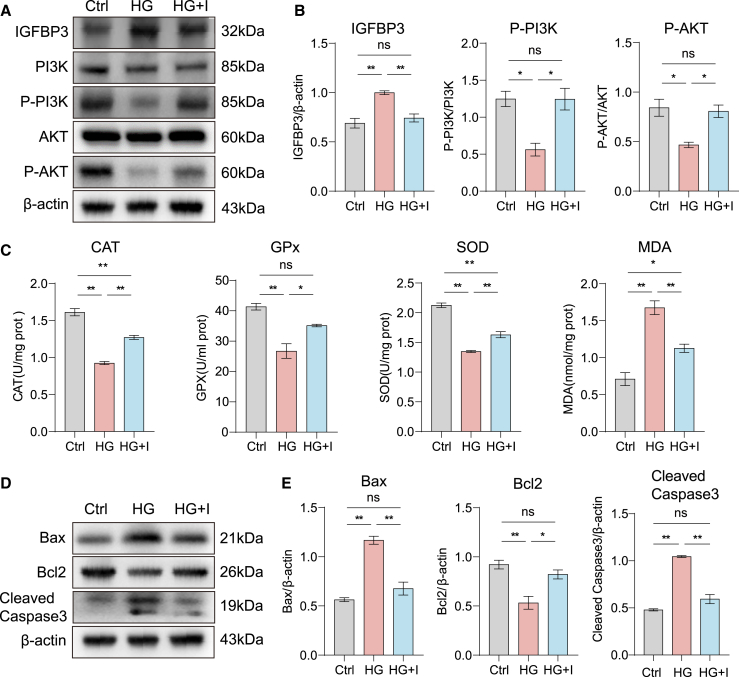
ICA alleviates oxidative stress and apoptosis in diabetic SD rats (A) Immunoblot analysis of the indicated proteins in the lens of SD rats in Ctrl, HG, and HG + I groups; 3 lenses were surgically harvested and pooled to extract protein for immunoblot assay. (B) Statistic analysis of (A). (C) The levels of CAT, GPx, SOD, and MDA in the lens of SD rats in Ctrl, HG, and HG + I groups; 3 lenses were surgically harvested and pooled to extract protein for immunoblot assay. (D) Immunoblot analysis of the apoptosis-relative proteins in the lens of SD rats in Ctrl, HG, and HG + I groups; 3 lenses were surgically harvested and pooled to extract protein for immunoblot assay. (E) Statistic analysis of (D). Data are representative of three independent experiments. The data are presented as the mean ± SEM. *n* = 15. ∗*p* < 0.05 and ∗∗*p* < 0.01.

Building on our previous findings demonstrating that ICA mitigates diabetic cataract progression in SD rats and elucidating its underlying mechanisms *in vitro*, we next validated these effects *in vivo*. ICA treatment significantly enhanced the phosphorylation of PI3K and AKT while reducing IGFBP3 expression in the lenses of diabetic rats (Figures 6A and 6B). Moreover, ICA increased the activities of CAT, GPx, and SOD, while reducing MDA levels (Figure 6C). Additionally, ICA modulated apoptotic signaling by downregulating Bax and caspase-3 and upregulating Bcl-2 expression (Figures 6D and 6E). Collectively, these results indicate that ICA interacts with IGFBP3 to exert protective effects against oxidative stress and apoptosis in diabetic SD rats via activation of the PI3K/AKT pathway.

## Discussion

Diabetic cataracts resulting from oxidative stress and apoptosis are one of the leading causes of blindness.^21^ Here, we investigated the effects of ICA on diabetic cataracts *in vivo* and *in vitro,* and revealed that ICA improved diabetic cataracts by inhibiting high glucose-induced oxidative stress and cell apoptosis. Numerous studies have demonstrated that high concentrations of mannitol do not significantly affect the viability of SRA01/04 cells, indicating that high glucose damages these cells through an osmolarity-independent mechanism. Consequently, a high-mannitol group was not included in our study.^22^

It has been reported that ICA can effectively treat eye diseases. Our previous research demonstrated that ICA alleviated uveitis by targeting peroxiredoxin 3 to regulate the polarization of retinal microglial cells toward the M1/M2 phenotype.^17^ In addition, ICA can improve diabetic retinopathy by promoting neurite outgrowth in retinal ganglion cells.^18^ Moreover, in Graves’ orbitopathy, ICA exerted a protective effect by inhibiting AMPK-dependent autophagy and lipogenesis in adipocytes.^23^ However, the role of ICA in diabetic cataracts has not been reported; our current study suggested that ICA can improve diabetic cataracts through inhibiting oxidant stress and cell apoptosis.

Both oxidative stress and cell apoptosis are closely related to high glucose-induced lens damage.^24^ High glucose stimulation increases ROS production, which mediates oxidative damage in lens cells. H_2_O_2_, the predominant ROS both in the aqueous humor and within lens cells, causes protein oxidation and aggregation, lipid peroxidation, and DNA damage, thereby increasing the oxidative burden in the lens.^25^ This would further increase the damage to LECs and lead to cataract development. Long-term exposure to oxidative stress and a variety of stress factors can cause cell apoptosis, ultimately resulting in the development of cataracts.^26^^,^^27^^,^^28^

Research has shown that the PI3K/AKT signaling pathway inhibited oxidative stress and apoptosis in LECs.^29^ Data showed that ICA protected the hearts of experimental rats from ischemia/reperfusion injury by activating the PI3K/AKT pathway and modulating cell apoptosis.^30^ Pretreatment with ICA reversed the decrease in phosphorylated PI3K/AKT expression and reduced cisplatin-induced apoptosis in myocardial cells.^31^ However, increased expression of IGFBP3 can also activate the PI3K/AKT pathway.^32^ In this study, we demonstrate that ICA binds to IGFBP3, thereby enhancing PI3K/AKT signaling and reducing oxidative stress and apoptosis in LECs.

IGFBP3 takes part in processes such as cell proliferation and apoptosis through AKT, p38MAPK, and Smad2/3 pathway. Studies in epithelial cells have shown that in the presence of Vascular Endothelial Growth Factor (VEGF) stimulation, IGFBP3 can reduce AKT phosphorylation to enhance pro-apoptotic responses in epithelial cells stimulated by VEGF.^33^ Therefore, ICA binding to IGFBP3 could activate the PI3K/AKT signaling pathway to suppress high glucose-induced oxidative stress and apoptosis. Moreover, both our experiments and other studies have confirmed that IGFBP3 may directly interact with PI3K to exert its effects.^34^^,^^35^ Critical molecular docking experiments established ICA-IGFBP3 binding. However, it remains to be further studied whether ICA specifically acts directly on the IGFBP3 protein, inhibiting the binding of IGFBP3 to IGF1 and activating downstream pathways, or if IGFBP3 acts through an IGF1-independent pathway. Moreover, it remains to be determined whether the increased expression of IGFBP3 in the aqueous humor is related to the systemic elevation of IGFBP3 following disruption of the blood-aqueous barrier. Furthermore, the expression levels of IGFBP3 in the aqueous humor differ among patients. In the future, this has potential to serve as an effective biomarker for diagnosing different types of cataracts, with promising prospects.

### Limitations of the study

There were several limitations in this study. Although ICA has demonstrated good therapeutic effects in treating diabetic cataract, there remain some issues regarding its administration and safety. Current studies indicate that ICA exhibits low oral bioavailability in humans, mainly due to insufficient intestinal absorption and significant first-pass metabolism. Moreover, ICA’s physicochemical properties limit its permeability across the blood-aqueous barrier, preventing rapid attainment of therapeutic intraocular concentrations and thus compromising clinical efficacy. Existing data suggest that ICA has relatively low overall toxicity, and its impact on liver and kidney functions remains within controllable limits; however, prolonged or high-dose usage might still pose a slight risk of toxicity. Therefore, future research should focus on optimizing drug delivery systems, such as using local injections or nanotechnology-based strategies, to improve intraocular delivery efficiency and bioavailability, as well as further refine safety assessments.

## Resource availability

### Lead contact

Requests for further information and resources should be directed to and will be fulfilled by the lead contact, Ke Hu (cqhuke@hospital.cqmu.edu.cn).

### Materials availability

This study did not generate new unique reagents.

### Data and code availability

• Any additional information required to reanalyze the data reported in this paper is available from the lead contact upon request.
• This paper does not report original code.
• Any additional information required to reanalyze the data reported in this paper is available from the lead contact upon request.

## Acknowledgments

This work was supported by 10.13039/501100001809National Natural Science Foundation of China (grant nos. 82371098 and 32200711), the Project Foundation of Chongqing Science and Technology Commission of China (grant nos. CSTC2021jscx-gksb-N0017 and cstc2021jcyj msxmX0967), Chongqing Talent Plan “Contract program” (grant no. cstc2022ycjh-bgzxm0121), Chongqing Science and Health Joint Key Project (grant no. 2024ZDXM033), Chongqing Young and Middle-aged High-end Talent Project (grant no. 2024GDRC005), and Beijing Municipal Public Welfare Development and Reform Pilot Project for Medical Research Institutes (PWD&RPP-MRI and JYY2023-6). The authors thank all participants who participated in this study at the Chongqing Medical University and Capital Medical University.

## Author contributions

K.H., S.H., and S. Zheng initiated and supervised the project. Y.W. and W.Y. designed the experiments, analyzed the data, and wrote the paper. Y.W. and H.Z. performed the experiments. X.L., X.G., Y.X., F.H., B.C., C.W., and H.F. helped with some experiments. S. Zheng and W.W. provided and analyzed the clinical samples. S. Zeng, F.C., H.Y., S.G., and M.T. helped analyze the data and revised the manuscript. K.H. is the guarantor of this work and, as such, had full access to all the data in the study and takes responsibility for the integrity of the data and the accuracy of the data analysis.

## Declaration of interests

The authors declare no competing interests.

## STAR★Methods

### Key resources table

**Table:** 

REAGENT or RESOURCE	SOURCE	IDENTIFIER
**Antibodies**		

IGFBP3 Polyclonal antibody	Proteintech	Cat No. 10189-2-AP
BAX Monoclonal antibody	Proteintech	Cat No. 60267-1-Ig
BCL2 Monoclonal antibody	Proteintech	Cat No. 60178-1-Ig
Cleaved Caspase 3 Monoclonal antibody	Proteintech	Cat No. 68773-1-Ig
PI3 Kinase p85 (19H8) Rabbit mAb	Cell Signaling Technology	Cat No. #4257
Akt (pan) (C67E7) Rabbit mAb	Cell Signaling Technology	Cat No. #4691
Phospho-Akt (Ser473) (D9E) XP® Rabbit mAb	Cell Signaling Technology	Cat No. #4060
Phospho-PI3K p85 (Tyr458)[Tyr467]/p55 (Tyr199) Antibody	Affinity	Cat No. AF3242
beta Actin Antibody	Affinity	Cat No. AF7018

**Chemicals, peptides, and recombinant proteins**		

Icariin	Shanghai Yuanye Biotechnology	Cat No. 489-32-7
Glutathione Peroxidase (GSH-PX) assay kit	Nanjing Jiancheng Bioengineering Institute	Cat No. A005-1-2
Catalase (CAT) assay kit	Nanjing Jiancheng Bioengineering Institute	Cat No. A007-1-1
Superoxide Dismutase (SOD) assay kit	Nanjing Jiancheng Bioengineering Institute	Cat No. A001-3-2
Malondialdehyde (MDA) assay kit	Nanjing Jiancheng Bioengineering Institute	Cat No. A003-1-2
Annexin V-FITC Apoptosis Detection Kit	Beyotime	Cat No. C1062L
Reactive Oxygen Species Assay Kit	Beyotime	Cat No. S0033S
Enhanced Cell Counting Kit-8	Beyotime	Cat No. C0041
Human IGFBP3 assay ELISA kit	Jiangsu Meimian	Cat No. 25097
LY294002	MCE	Cat No. HY-10108

**Experimental models: Cell lines**		

SRA01/04 Human Lens Epithelial Cells	BIOESN	Cat No. BES12113HC

**Experimental models: Organisms/strains**		

SD rat	Experimental Animal Center of Chongqing Medical University	Cat No. SD

**Software and algorithms**		

IGFBP-3 protein structure	UniProt	https://www.uniprot.org/
Icariin structure	pubchem	https://pubchem.ncbi.nlm.nih.gov/

### Experimental model and study participant details

#### Establishment of the STZ-induced diabetic SD rats model

In this study, a cohort of 45 male Sprague-Dawley rats, aged 8 weeks and weighing between 200 to 250 grams, was utilized. Rats were singly housed under standard pathogen-free conditions (12 h light/12 h dark) with *ad libitum* access to food and water. The experiments were approved by the Ethics Committee of Chongqing Medical University (IACUC-CQMU-2023-0350) and conducted according to institutional guidelines. Rats were fasted for 16 h, and 30 rats (diabetes mellitus group) were injected intraperitoneally with freshly prepared Streptozotocin (STZ, Sigma Chemical Co, St Louis, MO, USA) (65 mg/kg) and 15 rats (control group) injected vehicle (0.1 mol/L citrate-phosphate buffer, pH 4.5) according to the ref.^36^ 72 h post-injection of streptozotocin or vehicle, blood glucose levels were assessed. Glucose concentrations were determined from tail vein blood samples using a glucometer, and only rats exhibiting fasting hyperglycemia (≥16.7 mmol/L) were categorized into the DM group (type 1 diabetes model). A total of 30 rats developed into diabetes were divided into 2 groups, they fed respectively with a 50:1 mix of normal saline and dimethyl sulfoxide (DMSO, placebo, N = 15) and ICA (20 mg/kg/d) dissolved in 2% DMSO (ICA, N = 15) for 12 weeks. Non-diabetic control rats received PBS by oral gavage and otherwise identical husbandry. Regularly monitor the blood glucose and body weight of the SD rats. At the end of 12 weeks, rats were euthanized, and lenses were harvested for histological and molecular study.

#### Human subject

This investigation adhered to the principles outlined in the Declaration of Helsinki. Aqueous humor specimens were collected from the First Affiliated Hospital of Chongqing Medical University, with the procurement process receiving approval from the hospital’s Ethics Committee (protocol number 2023-1). Participants provided written informed consent following a thorough explanation of the study’s objectives and methodology. Patients who met the following criteria were enrolled in the control group: (1) Postoperative aphykic eye requiring lens adjustment, (2) non-diabetes (Ctrl, n = 13, female = 7, male = 6). Patients who met the following criteria were enrolled in the age-related cataract group: (1) diagnosed as age-related cataract, (2) non-diabetes (ARC, n = 59, female = 42, male = 17). Patients who met the following criteria were enrolled in the diabetic cataract group: (1) diagnosed as diabetes, Fasting blood glucose ≥ 7.0 mmol/L (2) diagnosed as cataract (DC, n = 28, female = 17, male = 11). Patients with concurrent ocular conditions other than the study focus, such as glaucoma, high myopia, or retinal pathologies, as well as those with a history of prior ocular interventions or severe systemic illnesses, including uncontrolled hypertension, were not included in this study.

Demographic and clinical data of the subjects, encompassing gender, diagnoses, ocular and metabolic disease history, overall medical background, preoperative intraocular pressure (IOP), best-corrected visual acuity (BCVA), and age at the time of cataract surgery, were extracted from the electronic medical records system. Furthermore, no gender differences have been reported for diabetic cataract.

#### Cell culture and treatment

The SRA01/04 human lens epithelial cell line (Cat No. BES12113HC) has been authenticated by BIOESN. The cell line underwent mycoplasma contamination testing and were confirmed to be contamination-free. It was propagated in DMEM supplemented with 10% FBS under incubation conditions of 5% CO2 at 37°C. Experiments were conducted using cells that had reached 80–90% confluence, which were plated in various culture vessels including flasks, dishes, and plates. A control group (Ctrl) was established with cells cultured under standard conditions, while a high-glucose (HG) group was defined by cells subjected to 50 mM glucose for 48 h.^37^ The cells were treated with high glucose + ICA (20 μM) as HG + I group. cells and supernatant were collected for detection.

### Method details

#### Slit-lamp examination

Rats’ eyes were evaluated every other week using a Digital Slit Lamp Microscope (Digital slit lamp LS-7DE, Chongqing Sunkingdom Medical Instrument Co., Ltd., Chongqing, China) after dilating the pupils (Tropicamide Phenylephrine Eye Drops; Santen Pharmaceutical Co., Ltd., Osaka, Japan) by a trained observer who was blinded to the treatment. The severity of cataracts was graded in the form of a 5-grade scoring system as follows: 0, clear normal lens; 1, peripheral vesicle; 2, peripheral vesicle plus cortical opacity; 3, diffuse central opacity; and 4, mature cataract.^38^^,^^39^

#### Histopathology analysis

Lens tissues were fixed immediately after harvest in 10% neutral-buffered formalin for 24 h at room temperature, then dehydrated through a graded ethanol series (70%, 80%, 95%, and 100%, 1 h each), cleared in xylene (2 × 30 min), and infiltrated with molten paraffin at 60°C (2 × 1 h). Embedded specimens were sectioned at 4 μm on a rotary microtome and mounted on poly-L-lysine–coated slides before deparaffinization in xylene (2 × 10 min) and rehydration through descending ethanol concentrations (100%, 95%, 80%, 70%, 5 min each). Standard hematoxylin and eosin staining was performed by immersing sections in hematoxylin for 5 min, rinsing under tap water for 2 min, differentiating in 1% acid alcohol for 10 s, bluing in 0.2% ammonia water for 30 s, and staining in eosin for 1 min, followed by rapid dehydration (95% and 100% ethanol) and clearing in xylene prior to mounting with DPX. Histological evaluation was carried out using an light microscope at 200× and 400× magnifications, with digital images captured via a camera.

#### Cell viability

SRA01/04 cells were cultured to 80% confluence, then trypsinized and counted with a hemocytometer. Cells were plated at 5,000 cells per well in 100 μL of complete DMEM in 96-well plates and allowed to adhere for 24 h. Following attachment, cells were treated with ICA at final concentrations of 0, 10, 20, 30, 40, or 50 μM (diluted from a 20 mM stock in DMSO; vehicle concentration ≤0.1%) for 48 h. After treatment, 10 μL of Cell Counting Kit-8 (CCK-8; Dojindo) reagent was added to each well, and plates were incubated at 37°C with 5% CO_2_ for 90 min. Absorbance at 450 nm was recorded using a microplate reader, with blank wells (medium plus CCK-8 without cells) used for background subtraction.

#### Analysis of oxidative stress indicators

The levels of CAT, GPx, SOD, and MDA in SRA01/04 cells and rats’ lens were measured using the CAT, GPx, SOD, and MDA detection kits and according to the operating instructions.

#### ROS staining

SRA01/04 cells in the logarithmic phase were seeded into 12-well plates at a density of 1 × 10^5^ cells per well, and experiments were initiated once the cells achieved 60% confluence. Following exposure to HG and ICA, the cells were incubated with 1 μM DCFH-DA in the dark at 37°C for 30 min. The resultant ROS levels were quantified by measuring the fluorescence intensity using an inverted fluorescence microscope (Leica DM, Germany).

#### Elisa

IGFBP3 protein levels in human aqueous humor were quantified using an ELISA kit. Aqueous humor samples stored at −80°C, were thawed at 4°C. Serial dilutions of standards and samples were prepared in ice-cold PBS to maintain protein stability. Pre-coated 96-well plates were loaded with 100 μL of standards or diluted samples in triplicate, followed by 1-h blocking with 5% BSA/PBS. After 2-h incubation at 37°C with primary antibodies, plates underwent five automated washes with PBS-T. HRP-conjugated secondary antibodies were added for 1-h incubation, followed by TMB substrate development (15 min, RT, shielded from light). Reactions were terminated, and absorbance was measured at 450 nm using a multimode reader.

#### Flow cytometry apoptosis detection

In accordance with the protocol of the Annexin V-FITC Apoptosis Detection Kit, the apoptotic rate among SRA01/04 cells across experimental groups was assessed. Post 48-h co-incubation with HG and ICA, the cells were harvested. They were then resuspended in 5 mL of pre-chilled PBS, centrifuged at 1000 rpm for 5 min, and subjected to apoptosis analysis using the kit. The cells were gently resuspended in 195 μL of Annexin V-FITC binding buffer. Subsequently, 5 μL of Annexin V-FITC and 10 μL of PI were added, mixed gently, and incubated at room temperature (20°C–25°C) in the dark for 10–20 min, followed by chilling on ice. The apoptotic rate of SRA01/04 cells was subsequently quantified using flow cytometry.

#### Western blotting assays

Equal volumes of protein samples were resolved on 10% Bis-Tris-polyacrylamide gels and transferred onto PVDF membranes. After blocking with 5% skim milk, the membranes were probed with primary antibodies at 4°C overnight. The antibodies used were sourced as follows: goat anti-rabbit IgG and goat anti-mouse IgG (1:5000, Proteintech, SA00001-2 and SA00001-1, respectively), IGFBP3 (1:200, Proteintech, 10189-2-AP), PI3K, AKT, and p-AKT (1:1000, Cell Signaling Technology, #4257, #4691, and 1:2000, #4060, respectively), Bax, Bcl-2, and Cleaved Caspase 3(1:1000, Proteintech, Cat No. 60267-1-Ig, Cat No. 60178-1-Ig, Cat No. 68773-1-Ig, respectively) and from Affinity, p-PI3K and β-actin (1:500, AF3242, and 1:5000, AF7018, respectively). Membranes were then incubated with HRP-conjugated anti-rabbit or anti-mouse IgG secondary antibodies for 1 h and visualized using ECL Plus detection reagents. Protein band intensities were quantified with ImageJ software and normalized against β-actin levels.

#### Molecular docking

The human IGFBP-3 protein structure (UniProt ID: P17936) was acquired from AlphaFold predictions via UniProt. Icariin (PubChem CID: 5318997) was retrieved from PubChem and energy-optimized using Chem3D to generate a 3D .pdb file. Both structures were processed in AutoDock Tools for hydrogen addition and charge assignment, then converted to pdbqt format. A 126 Å^3^ docking box was centered on the protein’s functional domain. Molecular docking was executed with AutoDock Vina, followed by PyMOL analysis of hydrogen-bonding residues and 3D interaction visualization.

#### Molecular dynamics simulations

A 200 ns molecular dynamics simulation was conducted using GROMACS 2020.6 with CHARMM36 and Amber GAFF force fields for the protein and ligand, respectively. The system was solvated in a cubic box (edge distance ≥1.0 nm) with water (1 g/cm^3^) and 0.15 mol/L Na^+^/Cl^−^ for neutralization. Energy minimization (steepest descent) resolved atomic conflicts, followed by NVT (300 K, 100 ps) and NPT (1 bar, 100 ps) pre-equilibration. Production simulations under isothermal-isobaric conditions (300 K, 1 bar) employed the leapfrog Verlet algorithm. Post-simulation, structures were aligned to eliminate system motion, enabling RMSD analysis of internal conformational changes.

#### LC-MS/MS analysis

Tryptic peptides were dissolved in solvent A (0.1% formic acid, 2% acetonitrile in water) and directly loaded onto a homemade reversed-phase analytical column (25 cm length, 100 μm i.d.). The mobile phase comprised solvent A and solvent B (0.1% formic acid in acetonitrile). Peptide separation was achieved using the following gradient: 0–14 min, 6%–24% B; 14–16 min, 24%–35% B; 16–18 min, 35%–90% B; and 18–20 min, 90% B, all at a constant flow rate of 500 μL/min on an Easy-nLC1000 UHPLC system (Bruker Daltonics). The separated peptides were then introduced into a capillary source for analysis using timsTOF Pro mass spectrometry, with an electrospray voltage of 1.75 kV. Both precursor and fragment ions were analyzed using the TOF detector, and the timsTOF Pro operated in data-independent parallel accumulation serial fragmentation (dia-PASEF) mode. The full MS scan range was set from 300 to 1500 m/z, with 20 PASEF MS/MS scans acquired per cycle. The MS/MS scan range was established at 400–850 m/z, and the isolation window was set to 7 m/z.

The DIA data were processed using the DIA-NN search engine (v. 1.8). Tandem mass spectra were searched against the Homo sapiens database (Homo_sapiens_9606_SP_20230103.fasta, 20,389 entries) concatenated with a reverse decoy database. Trypsin/P was designated as the cleavage enzyme, allowing for up to one missed cleavage. Fixed modifications included N-terminal methionine excision and carbamidomethylation of cysteine residues. The false discovery rate (FDR) was adjusted to <1%.

#### Surface plasmon resonance assay

The binding affinity was assessed using a Biacore T200 instrument (Cytiva). IGFBP3 was prepared at a concentration of 25 μg/mL in sodium acetate buffer (pH 4.0, 10 mM) and immobilized on the CM5 sensor chip’s channel two, with channel one serving as a reference control. ICA was prepared in the mobile phase buffer (10 mM HEPES, pH 7.4, 150 mM NaCl, 3 mM EDTA, 0.05% P20) to achieve the desired concentrations. The sample was then introduced to the chip for a 120-s association phase, followed by a 180-s dissociation phase, with the running buffer acting as the blank control.

#### Predicting the targets of diabetic cataract

GeneCards database (https://www.genecards.org/) were used together the information on diabetic cataract-associated target genes. GeneCards is a comprehensive database of functions involving proteomics, genomics, and transcriptomics. The keywords ‘diabetic cataract’ were utilized to screen the diabetic cataract-associated targets. The names of targets were collected from GeneCards, which provided information about the protein targets, the corresponding ID of each target and the targeted disease. The common targets of ICA and diabetic cataract were then gathered as the core targets of ICA for diabetic cataract.

#### KEGG pathway enrichment analysis

Pathway analysis was conducted utilizing the Kyoto Encyclopedia of Genes and Genomes (KEGG) database through the R package ‘ClusterProfiler’. The enrichment analysis was set with a statistical significance threshold of p < 0.05.

### Quantification and statistical analysis

#### Statistical analysis

Data are expressed as mean ± SEM and were analyzed using SPSS version 20.0. For comparisons among multiple groups, one-way ANOVA followed by post-hoc Least Significant Difference (LSD) correction was employed. Clinical and pathological scoring data were assessed using the Kruskal-Wallis test for multiple group comparisons and the Mann-Whitney U test for pairwise analyses. Statistical significance was set at *P* < 0.05 (∗*P* < 0.05, ∗∗*P* < 0.01).
