# Supplementary material for: Icariin interacts with IGFBP3 to alleviate diabetic cataract through PI3K/AKT signaling pathway
Source: iScience. 2025 May 30;28(7):112796. doi: 10.1016/j.isci.2025.112796 (PMC12226389; doi:10.1016/j.isci.2025.112796)
Supplement: Document S1. Figures S1–S4 [file mmc1.pdf]

iScience, Volume *28*

Supplemental information

Icariin interacts with IGFBP3 to alleviate diabetic cataract through PI3K/AKT signaling pathway

Yakun Wang, Wenxian Yang, Hangjia Zuo, Shuhao Zeng, Xianyang Liu, Fan Cao, Hui Yang, Shangze Gao, Meng Tian, Xiang Gao, Yongguo Xiang, Fanfan Huang, Baorui Chu, Chao Wu, Hui Feng, Wenjuan Wan, Shijie Zheng, Shengping Hou, and Ke Hu

Supplementary Figures

A

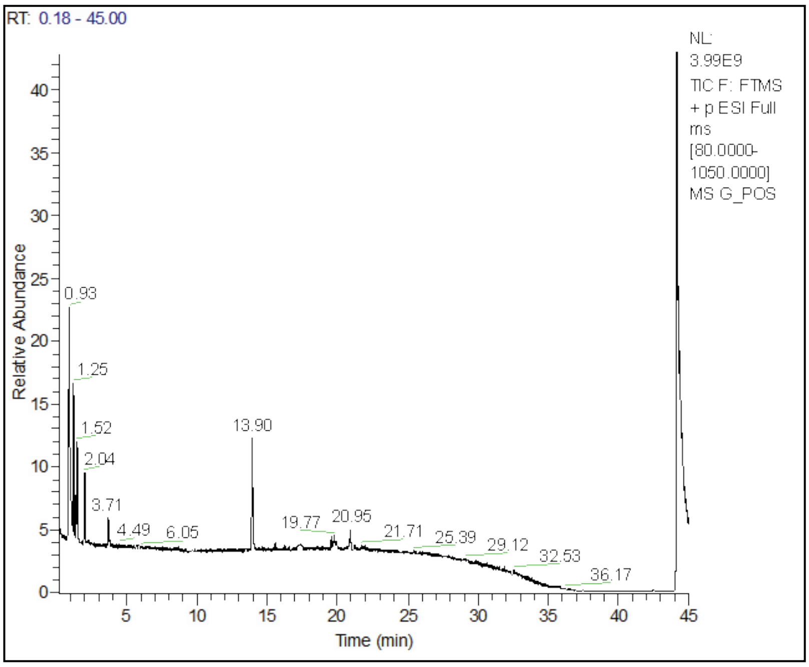

B

NO.	Name	Reference m/z	Formula	RT (min)	Height	Area	Adduct	Ontology
1	Pyruvate	89.51	C3H4O3	0.102	125909648	1028179581	[M+H]+	Alpha-keto acids and derivatives
2	Icariin	677.24402	C33H40O15	13.896	181783430	960661072	[M+H]+	Flavonoid-7-O-glycosides
3	4-hydroxy-4-(pyridin-2-yl)butan-2-one	166.09	C9H11NO2	2.041	167006678	791155312	[M+NH4]+	Pyridines and derivatives
4	Theobromine	203.05	C7H8N4O2	0.945	108719407	637996514	[M+Na]+	Xanthines
5	L-Isoleucine	132.10191	C6H13NO2	1.44	110621588	354387731	[M+H]+	Isoleucine and derivatives

Figure S1. The concentration of icariin (ICA) in rat aqueous humor. A.) Total ion current chromatograms (TICs) of SD rats aqueous humor recorded in the positive modes. Identification information of SD rats aqueous humor. B.) Identification information of SD rats aqueous humor.

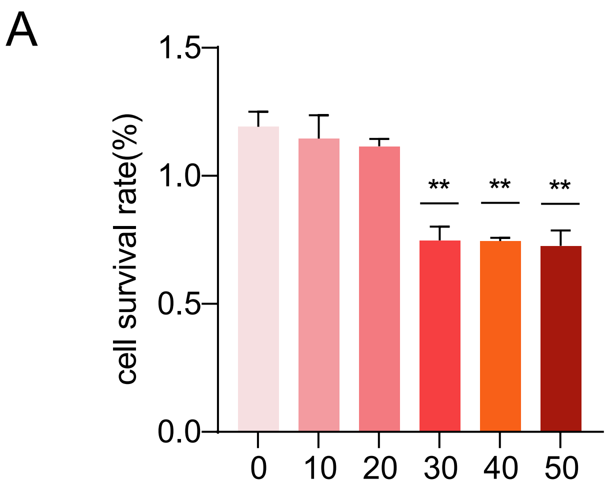

Figure S2. Impact of icariin (ICA) on cell survival rate. A.) Cell viability assay of the CCK-8 kit on SRA01/04 cells treated with different doses of icariin for 48 h

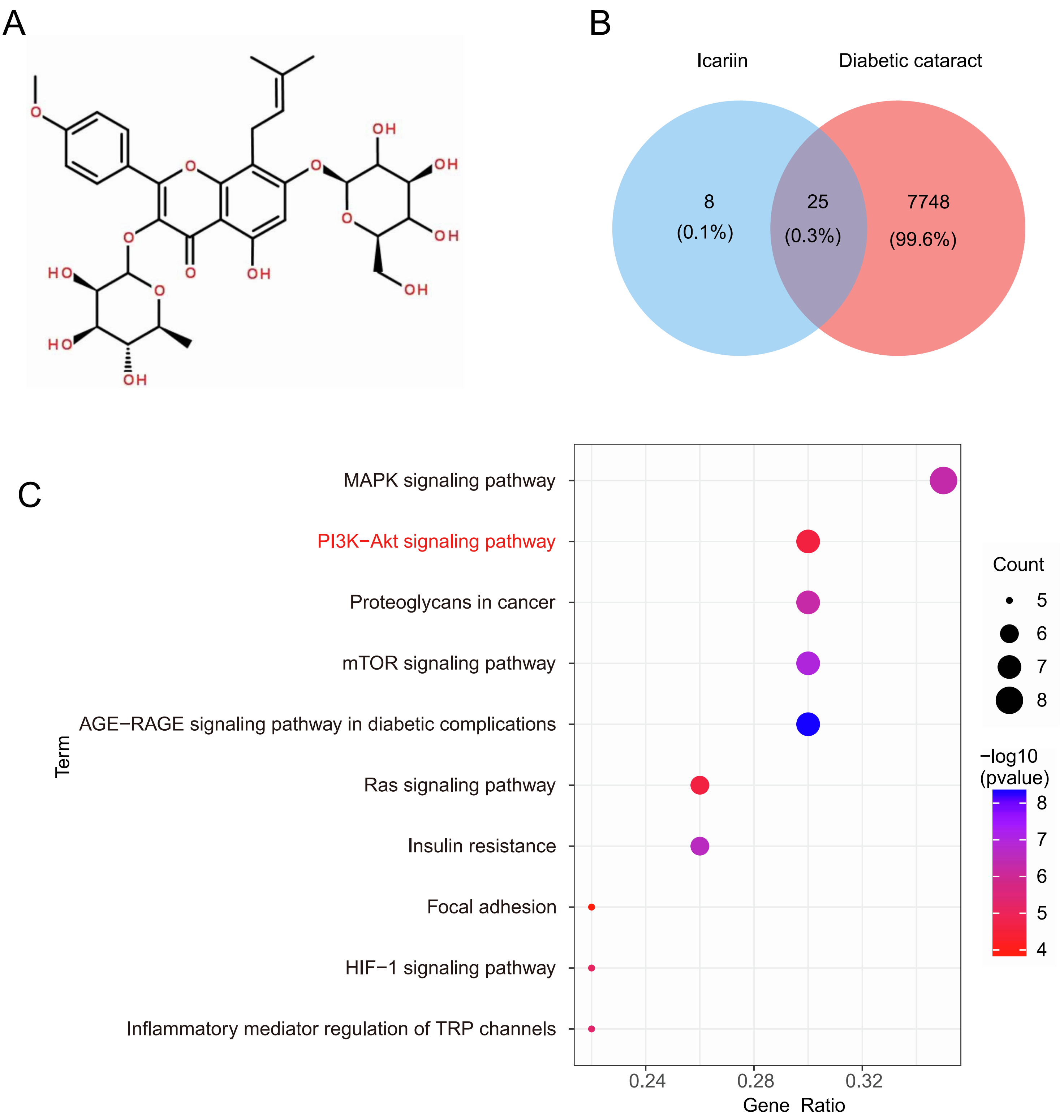

Figure S3. Network Pharmacology Analysis of Icariin(ICA). A.) The structural formula of icariin. B.) Wayne diagram of disease target and drug targets among icariin and diabetic cataract. C.) KEGG pathways.

A

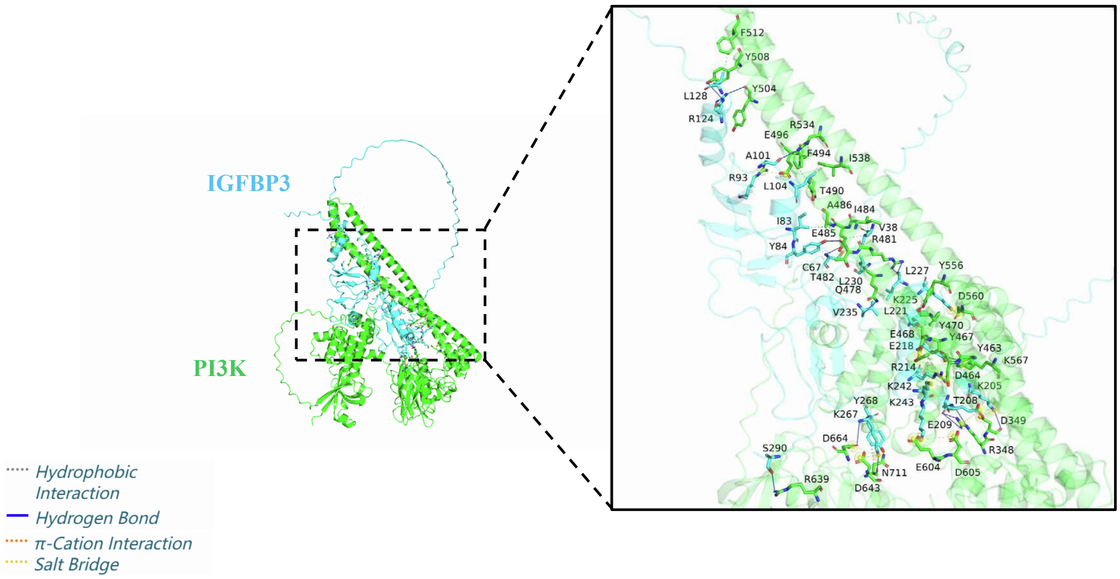

Figure S4. IGFBP3 interact with PI3K. A.) 3D Interaction Diagram of IGFBP3 and PI3K.
